# Cytomegalovirus anogenital ulcers in an immunosuppressed patient

**DOI:** 10.1016/j.jdcr.2025.01.046

**Published:** 2025-03-28

**Authors:** Karla L. Valdes Morales, Marc Bornstein, Claire E. Hannah, Thomas Vazquez, Corinne Rauck, Olaf Rodriguez, Rosalie Elenitsas, Alyssa Ammazzalorso, Ilyas Sahrish, Blair C. Weikert, Emily Baumrin, Meera Sivendran, Amy Forrestel, Misha Rosenbach

**Affiliations:** a Department of Dermatology, Hospital of the University of Pennsylvania, Philadelphia, Pennsylvania; b Perelman School of Medicine, University of Pennsylvania, Philadelphia, Pennsylvania; c Department of Dermatopathology, Hospital of the University of Pennsylvania, Philadelphia, Pennsylvania; d Division of Infectious Disease, Hospital of the University of Pennsylvania, Philadelphia, Pennsylvania

**Keywords:** anogenital ulcers, cytomegalovirus, HIV, immunosuppression

## Introduction

Cytomegalovirus (CMV) is a common opportunistic infection in immunocompromised patients, including those with advanced human immunodeficiency virus (HIV). Though CMV infections in individuals living with HIV have decreased in the era of antiretroviral therapy, localized or disseminated disease can still occur when CD4+ counts are lower than 50 cells/uL.^1^ The manifestations of clinical disease due to CMV are broad and can include retinitis, pneumonitis, hepatitis, encephalitis, and gastrointestinal disease. Tissue-invasive cutaneous involvement from CMV is rare and typically manifests with anogenital ulcerations. One of the most common infectious causes of anogenital ulcerations in immunocompromised patients is herpes simplex virus (HSV).^2^ In patients with advanced HIV, anogenital ulcerations will often test positive for both HSV and CMV by viral PCR, but resolve with HSV-directed antivirals alone, suggesting that CMV is often a bystander rather than playing a pathogenic role.^2^^,^^3^ We present a case of treatment-refractory, HSV-positive genital ulcers in an HIV-positive immunosuppressed patient, who was later diagnosed with CMV coinfection and responded to CMV-directed therapies.

## Case report

A woman in her 40s with a 7-year history of HIV infection treated with dolutegravir, tenofovir alafenamide, and emtricitabine was hospitalized for a recent diagnosis of Hodgkin’s lymphoma complicated by hemophagocytic lymphohistiocytosis. She was treated with dexamethasone, cyclophosphamide, doxorubicin, vinblastine, and dacarbazine. She was on prophylactic dosing of oral acyclovir (400 mg twice daily). At the time of admission, her CD4 count was 11 cells/uL, though with a CD4 percentage of 28% (normal range 32% to 56%). She had an undetectable HIV viral load with a peak viral load of 1610 IU/mL 2 months prior. Her hospital stay was complicated by acute renal failure requiring intermittent hemodialysis, *Enterococcus cecorum* and *Staphylococcus epidermidis* polymicrobial bacteremia, as well as refractory oral intolerance, vomiting, and diarrhea due to chemotherapy side effects and *Clostridium difficile* pancolitis.

**Fig 1:**
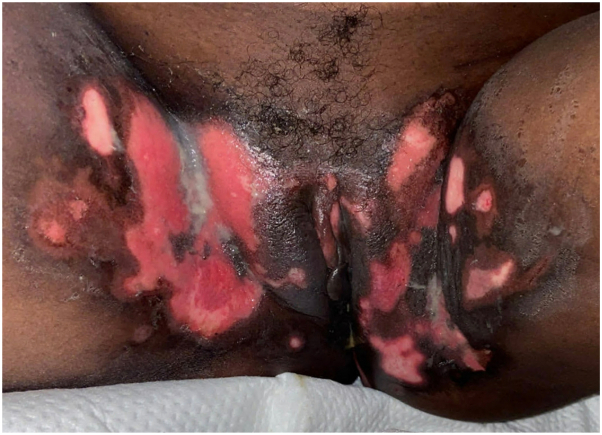
Anogenital ulcers in patient with HIV.

**Fig 2:**
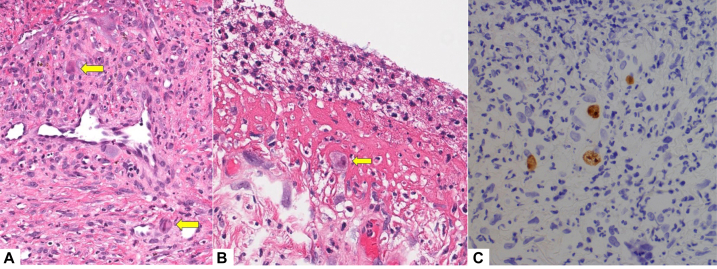
High power showing eosinophilic inclusion bodies (*arrows*) in dermis (**A**, H&E 200×) and at the base of the ulcer (**B**, H&E 400×). Immunoperoxidase staining for cytomegalovirus highlights cells in the superficial dermis (**C**, 400×).

During her hospital stay, she developed painful anogenital ulcerations. Physical evaluation revealed multiple ulcers with well-demarcated borders and a clean base in the vulvar and perianal regions, inguinal folds, and proximal inner thighs (Fig 1). Viral swab of the ulcers was positive for HSV-2 by polymerase chain reaction (PCR). She was initiated on treatment-dose IV acyclovir dosed according to renal function (5-10 mg/kg daily), as well as topical cidofovir 1% ointment. Despite 4 weeks of treatment, the ulcers continued to progress leading to concern for acyclovir-resistant anogenital HSV infection. Acyclovir resistance testing was sent but ultimately could not be performed due to inadequate growth on viral culture. Repeat viral swab was performed and was negative for HSV, but positive for CMV by PCR. Quantitative serum CMV level by PCR was elevated at 5140 IU/mL. She was treated empirically for cutaneous CMV with oral valganciclovir adjusted for renal function (450-900 mg twice daily). After 1 week on valganciclovir, ulcerations continued to progress, so skin biopsy was performed to clarify the etiology. Histology demonstrated viral cytopathic changes and intracellular inclusions highly suggestive of CMV infection, corroborated by positive CMV immunostaining (Fig 2); immunostains for HSV were negative.

**Fig 3:**
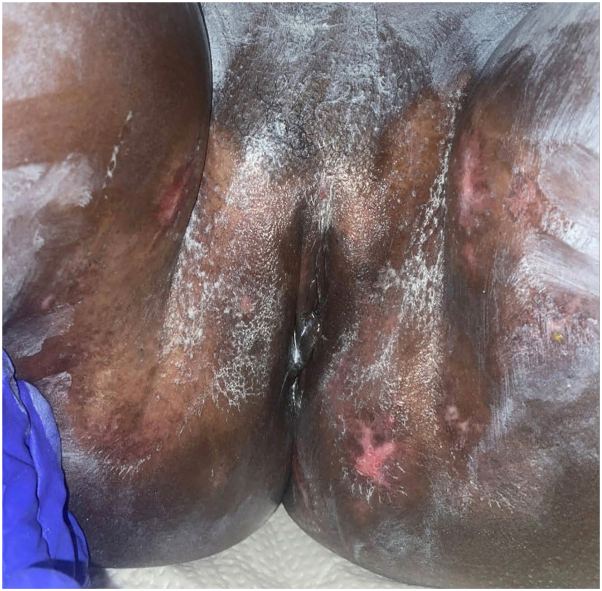
Follow-up at 2 months of treatment.

Given the presence of biopsy-confirmed, tissue-invasive cutaneous CMV in the setting of CMV viremia and progressive disease on valganciclovir, she was switched to IV ganciclovir (2.5 mg/kg twice daily). Ophthalmology evaluation was negative for CMV retinitis. Despite persistent abnormal liver function tests, diarrhea, and biopsy-proven cutaneous involvement, no evidence of CMV in other organs was confirmed. Genital ulcerations started improving within 1 week of IV ganciclovir and nearly resolved after 2 months of treatment (Fig 3). Serum CMV level by PCR progressively declined to undetectable levels. After 2.5 months of IV therapy, she was switched to oral valganciclovir, but treatment was discontinued due to worsening neutropenia. Two weeks later, vulvar ulcers recurred, with a positive viral swab for CMV by PCR (negative for HSV) and peak CMV viral load of 16,700 IU/mL. Intravenous ganciclovir was resumed with rapid improvement of ulcers and was then transitioned to oral valganciclovir. After 1 month of treatment, her CMV viral load was <35 IU/mL. She currently continues oral valganciclovir with control of skin lesions.

## Discussion

We present the case of a patient with recalcitrant genital ulcerations in the setting of mixed-etiology immunodeficiency (HIV and Hodgkin’s lymphoma treated with multiagent chemotherapy), which were initially HSV+ and CMV+ but persisted despite acyclovir with near complete resolution of disease following treatment with IV ganciclovir, suggesting dual-viral cutaneous infection with HSV and CMV. True tissue-invasive cutaneous CMV is extremely rare and most often manifests with anogenital ulcerations. Maculopapular or pustular rash, petechiae, and purpura have also been reported.^4^ Anogenital ulcerations are the most common manifestation of cutaneous disease due to high rates of viral shedding in the feces of immunosuppressed patients and prolonged contact between skin and feces.^2^^,^^5^

CMV PCR positivity is often considered a bystander in anogenital ulcers in immunosuppressed patients, as reactivation of the virus may be present in healthy skin in the setting of immunosuppression or CMV viremia.^3^ While skin swabs for CMV may demonstrate PCR positivity, this may be due to incidental low-level fecal shedding and is not on its own diagnostic for CMV-associated skin lesions. However, given the association of CMV with higher occurrence of non-AIDS defining events,^6^^,^^7^ consider testing for CMV upfront, as co-infections are common in immunocompromised patients, particularly those with HIV+. Our patient had a viral swab that was HSV-2 positive initially, which then cleared after treatment with acyclovir; however, her anogenital lesions persisted, leading to additional testing confirming CMV infection.

A number of recent case reports, including the case described herein, demonstrate that clinically significant cutaneous CMV disease should be considered in immunosuppressed patients who are not responding to treatment for HSV.^7^^,^^8^ Biopsy is instrumental to confirm the presence of virus in the tissue. Treatment guidelines are not yet established for cutaneous CMV lesions; however, we and others report the successful use of ganciclovir or valganciclovir.^4^^,^^8^^,^^9^ Intravenous ganciclovir is indicated in cases of severe disease, as well as cases where absorption of oral valganciclovir is impaired due to vomiting or diarrhea.

To date, no prophylactic antiviral therapy is recommended for the prevention of CMV end-organ disease in patients with HIV.^1^^,^^10^ CMV viremia has been associated with a higher occurrence of non–AIDS-defining events, which can lead to increased morbidity in affected patients.^6^ Our case highlights the immediate recurrence of CMV viremia and cutaneous lesions following the discontinuation of treatment in a patient with advanced HIV further immunosuppressed by chemotherapy, raising the question of whether preventive or maintenance CMV therapy is necessary for these patients.

Persistent, refractory anogenital ulcerations in the setting of immunosuppression should raise concern for CMV and prompt workup for tissue-invasive disease. Biopsy remains the gold standard for assessing the presence of tissue-invasive disease. Further research is needed to determine the clinical utility of viral swabs for CMV PCR in diagnosing cutaneous CMV disease.

## Conflicts of interest

None disclosed.

## References

[1] Panel on guidelines for the prevention and treatment of opportunistic infections in adults and adolescents with HIV. Guidelines for the prevention and treatment of opportunistic infections in adults and adolescents with HIV. National Institutes of Health, Centers for Disease Control and Prevention, HIV Medicine Association, and Infectious Diseases Society of America. https://clinicalinfo.hiv.gov/en/guidelines/adult-and-adolescent-opportunistic-infection.

[2] Chandler D.J., Walker S.L. (2023). HIV and skin infections. Clin Dermatol.

[3] Daudén E., Fernández-Buezo G., Fraga J., Cardeñoso L., García-Díez A. (2001). Mucocutaneous presence of cytomegalovirus associated with human immunodeficiency virus infection: discussion regarding its pathogenetic role. Arch Dermatol.

[4] Drozd B., Andriescu E., Suárez A., De la Garza Bravo M.M. (2019). Cutaneous cytomegalovirus manifestations, diagnosis, and treatment: a review. Dermatol Online J.

[5] Moscarelli L., Zanazzi M., Rosso G. (2011). Can skin be the first site of CMV involvement preceding a systematic infection in a renal transplant recipient?. NDT Plus.

[6] Lichtner M., Cicconi P., Vita S. (2014). Cytomegalovirus coinfection is associated with an increased risk of severe non–AIDS-defining events in a large cohort of HIV-infected patients. J Infect Dis.

[7] Hodowanec A.C., Lurain N.S., Krishnan S., Bosch R.J., Landay A.L. (2019). Increased CMV IgG antibody titer is associated with non-AIDS events among virologically suppressed HIV-positive persons. Pathog Immun.

[8] Tanaka A., Yamashita C., Hinogami H., Shirai H., Matsuura A. (2020). Cytomegalovirus-induced vasculopathy and anogenital skin ulcers in a patient with multiple myeloma. JAAD Case Rep.

[9] Shree K.K., Somashekar S., Loganathan E. (2022). Cytomegalovirus induced genital ulcer in human immunodeficiency virus positive patient. Indian J Sex Transm Dis AIDS.

[10] Wohl D.A., Kendall M.A., Andersen J. (2009). Low rate of CMV end-organ disease in HIV-infected patients despite low CD4+ cell counts and CMV viremia: results of ACTG protocol A5030. HIV Clin Trials.

